# Recent advances in innovative strategies for enhanced cancer photodynamic therapy

**DOI:** 10.7150/thno.54227

**Published:** 2021-01-15

**Authors:** Tingting Hu, Zhengdi Wang, Weicheng Shen, Ruizheng Liang, Dan Yan, Min Wei

**Affiliations:** 1 State Key Laboratory of Chemical Resource Engineering, Beijing Advanced Innovation Center for Soft Matter Science and Engineering, Beijing University of Chemical Technology, Beijing 100029, P. R. China.; 2 Beijing Friendship Hospital, Capital Medical University, Beijing 100050, P. R. China.

**Keywords:** photodynamic therapy, light source, photosensitizers, tumor hypoxia

## Abstract

Photodynamic therapy (PDT), a non-invasive therapeutic modality, has received increasing attention owing to its high selectivity and limited side effects. Although significant clinical research progress has been made in PDT, the breadth and depth of its clinical application have not been fully realized due to the limitations such as inadequate light penetration depth, non-targeting photosensitizers (PSs), and tumor hypoxia. Consequently, numerous investigations put their emphasis on innovative strategies to overcome the aforementioned limitations and enhance the therapeutic effect of PDT. Herein, up-to-date advances in these innovative methods for PDT are summarized by introducing the design of PS systems, their working mechanisms and application examples. In addition, current challenges of these innovative strategies for clinical application, and future perspectives on further improvement of PDT are also discussed.

## Introduction

**Scheme 1:**
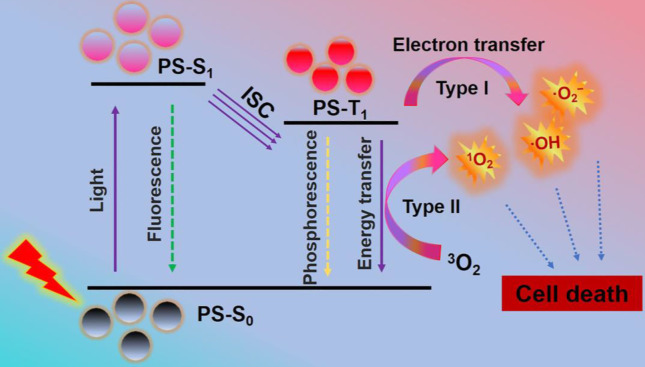
Mechanism of photodynamic reaction: PSs in S_0_ can transform into S_1_ by absorbing light. The S_1_ can change to T_1_ through ISC. The direct energy transfer from T_1_ to ^3^O_2_ or electron transfer between T_1_ and cellular substrates can produce ROS to induce cell death. Abbreviations: S_0_: ground state; S_1_: excited singlet state; T_1_: triplet state.

As a global medical problem in modern society, cancer is a serious threat to human health due to its high morbidity and mortality 1,2. Therefore, novel cancer therapies with few side effects and excellent curative effects are highly needed. Photodynamic therapy (PDT), a cancer therapy with clinical appeal, has received extensive attention owing to its high selectivity, minimally invasive nature, and limited side effects 3,4. Briefly, PDT adopts light-excited photosensitizers (PSs) to generate lethal reactive oxygen species (ROS), such as singlet oxygen (^1^O_2_), superoxide radicals (·O_2_^-^) and hydroxyl radicals (·OH). It works mainly through type I and type II mechanism (**Scheme 1**). Initially, PSs in ground state (S_0_) can transform into excited singlet state (S_1_ or ^1^PS*) by absorbing light. Then, the ^1^PS* can change to triplet state (T_1_ or ^3^PS*) through intersystem crossing (ISC) 30. In type II mechanism, the PDT effect is highly dependent on the oxygen (O_2_) content, since there is direct energy transfer from ^3^PS* to surrounding O_2_ to produce highly reactive ^1^O_2_ 5-7. While in type I mechanism, the ^3^PS* usually undergo electron transfer with cellular substrates in a physiological environment to produce toxic free radicals (e.g. ·OH and ·O_2_^-^) without O_2_ dependence. The resultant ROS can cause irreversible and permanent damage to cancer cells, and eventually lead to cell apoptosis and/or apoptosis, immune response, and microvascular damage 8-10.

Generally, light source, PSs and O_2_ are the three necessary components of PDT. With the continuous development of PSs and optical fiber-guided laser transmission systems, PDT has evolved from theoretical research to clinical research and expanded to imaging and treatment of various cancer and non-cancer diseases 11-15. However, like other therapies, the breadth and depth of PDT's efficacy have not been fully realized due to the limitations such as light penetration depth, inefficient PSs, targeted distribution, and tumor hypoxia 16-20. As PDT works only when the light reaches the target area, its therapeutic effect is quite limited to cancers in the skin areas or areas adjacent to the organ. In term of PSs, the difficulty of systemic administration is that PSs are generally easy to aggregate and lack targeting, thus limiting the clinical PDT efficacy. In addition, due to the excessive proliferation of cancer cells and insufficient blood supply in tumors, the O_2_ content in tumors is severely insufficient, resulting in a significant reduction of PDT efficacy. As a result, extensive research is being carried out to optimize viable PSs systems to overcome the aforementioned limitations.

**Figure 1:**
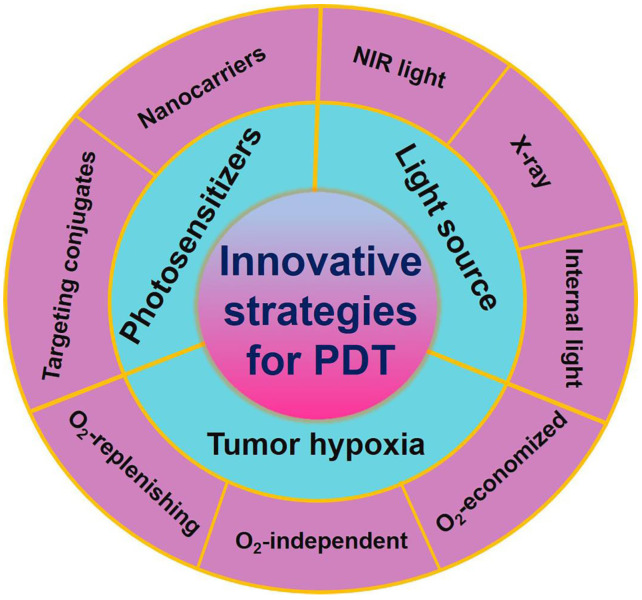
Schematic illustration of the innovative strategies for overcoming limitations (such as inadequate light penetration depth, non-targeting PSs, and tumor hypoxia) and enhancing the therapeutic effect of PDT. Abbreviations: PDT: photodynamic therapy.

Great efforts have been devoted to developing PDT and a number of strategies have been rationally proposed in recent years. Advances in PDT have been discussed a lot in previous reviews, and some of them emphasize on specific aspects, such as hypoxic tumor, tumor microenvironment (TME)-responsive PDT, types of PSs and their activation strategies, and the nanomaterials used in PDT. New techniques applied in PDT, such as ultrasound, microwaves and X-rays have also been reviewed 30,142-145. Nevertheless, little attention has been paid to the comprehensive and in-depth profile of recent strategies in PDT. In this review, we summarize and discuss the latest advancements and paradigms of PDT in cancer therapy by introducing the design of PS systems, their working mechanisms and application examples with a focus on the innovative strategies to improve the light penetration depth, ROS production efficiency, stability and targeted ability of PSs, and tumor hypoxia (**Figure 1**). At the end of the review, upcoming challenges and future perspectives of these innovative strategies (especially in clinical application) are discussed.

## Light Source

For PDT in cancer treatment, appropriate light source, adequate PSs, and abundant O_2_ are the three essential components required in tumor tissues 21,22. Among them, the choice of light source is particularly important because the light needs to be delivered homogeneously to the target area to ensure the therapeutic effect. Most PSs used for PDT have maximum absorption in the visible region (400-700 nm). To date, four types of laser light sources (argon-pumped lasers, metal-vapor-pumped lasers (Au- or Cu-vapor lasers), solid-state lasers (Nd:YAG lasers, Ho:YAG lasers, KTP:YAG/dye lasers), and diode lasers) and three non-laser light sources (lamp light, light-emitting diodes (LEDs), and daylight) have been tested in PDT 23-28. Some of them have been used clinically, such as a red light argon dye laser, Nd:YAG laser, a red light (635 nm) LED lamp, a 570-670 nm wavelength red light lamp, green light (520 nm), 420-nm blue light-emitting diode, daylight, etc. However, the penetration depth of these light sources is quite low (1-6 mm) for two reasons: (1) many endogenous chromophores in biological tissues, such as cytochromes and haemoglobin, can absorb visible light; (2) the heterogeneous structure of biological tissues can lead to light scattering, diffusing and disorienting, and thus significantly affects the photodynamic effect 30. As a result, near-infrared (NIR) light, X-ray, interstitial light, and internal light are employed in innovative strategies for deeper penetration depth 20,29. On this basis, recent research progress of PDT in light source will be discussed in detail in this section.

### NIR light

**Figure 2:**
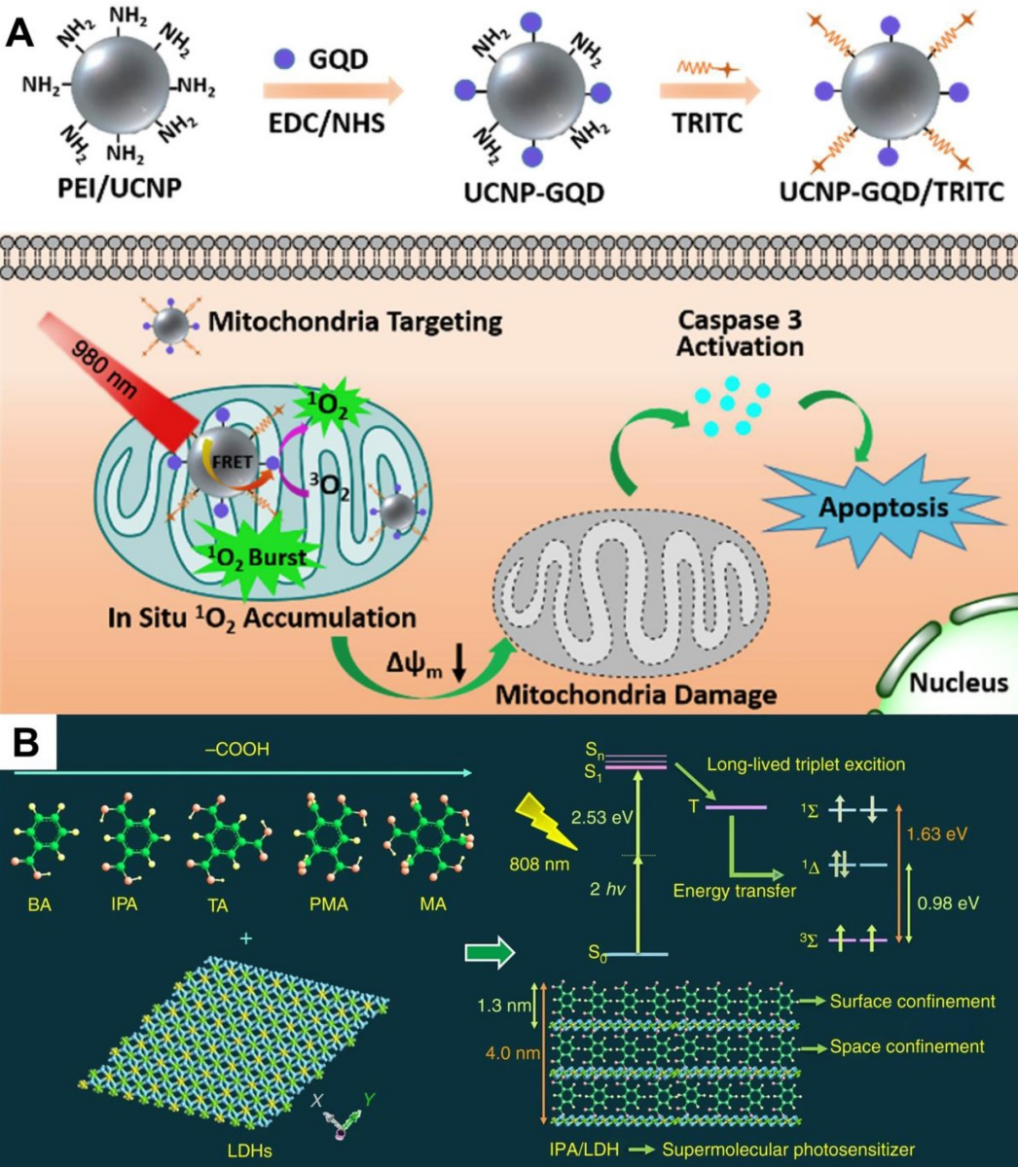
(**A**) Schematic diagram of UCNPs-GQD with mitochondria-targeting potency for high-efficient cells apoptosis upon laser irradiation. Reproduced with permission from Ref. 32, Copyright © 2017, Elsevier. (**B**) Schematic illustration of IPA/LDH nanohybrids as two-photon PSs for ^1^O_2_ generation under 808 nm NIR laser. Reproduced with permission from Ref. 33, Copyright © 2018, Nature Publishing Group. Abbreviations: UCNPs: upconversion nanoparticles; GQD: graphene quantum dot; IPA: isophthalic acid; LDH: layered double hydroxides; PSs: photosensitizers; ^1^O_2_: singlet oxygen; NIR: near-infrared.

It is generally known that NIR light (700-1350 nm), which can be divided into NIR I (700-1000 nm) region and NIR II (1000-1350 nm) region, is the 'optical window' of biological tissues. Compared with visible light, long-wavelength NIR light minimizes the degree of tissue scattering with a penetration depth exceeding 1 cm. The utilization of NIR light not only allows deeper tumor penetration but also reduces the phototoxicity on healthy tissues 20. Recently, in the work of Liu *et al.* 31, a NIR light-activated PDT nanosystem based on the combination of porphyrinic metal-organic frameworks (MOFs) and upconversion nanoparticles (UCNPs) was reported. The system was synthesized by growing porphyrinic MOFs on Nd^3+^-sensitized UCNPs to achieve Janus nanostructures. UCNPs could convert the low-energy NIR light to high-energy UV or visible light so as to excite porphyrinic MOFs to generate ROS. *In vitro* and *in vivo* tests certified the superior therapeutic effect of NIR-activated PDT. In another work, Zhang *et al.* 32 proposed a similar strategy that integrated rare-earth-doped UCNPs with graphene quantum dot (GQD) to form GQD-decorated UCNPs (UCNPs-GQD). Under NIR excitation, UCNPs could emit UV-vis light to excite the prominent ^1^O_2_ generation of GQD for highly efficacious PDT (**Figure 2A**). This upconversion technique seems quite promising in increasing penetration depth, however, it is limited by low energy conversion efficiency to a certain extent.

A recent breakthrough reported by Gao *et al.* 33 in NIR-activated PDT solved the above problem, and high-efficiency NIR-activated PSs were developed. This work assembled isophthalic acid (IPA, a type of room-temperature phosphorescence (RTP) compound) into the layered double hydroxides (LDHs) interlayer gallery, which formed an IPA/LDH nanohybrid (**Figure 2B**). In this PS system, the LDH monolayers provided space- and interface-confined microenvironments for IPA, facilitating the two-photon-induced generation of long-lived triplet exciton with a ^1^O_2_ quantum yield up to 0.74. Due to the excellent tissue penetration ability of 808 nm light, the IPA/LDH nanohybrid displayed superior anticancer performance with extremely low toxicity. This work provides a proof of concept that long-lived RTP compounds can be used as two-photon-activated PSs for NIR-activated PDT.

### X-ray

**Figure 3:**
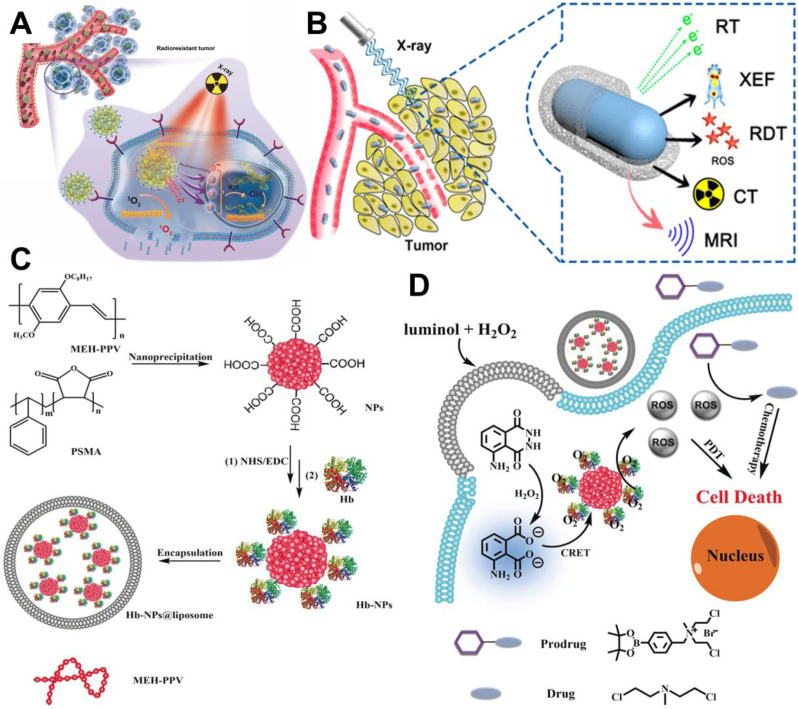
(**A**) Schematic representation of R-AIE-Au for fluorescence and CT imaging-guided X-ray-induced PDT. Reproduced with permission from Ref. 34, Copyright © 2019, Wiley-VCH Verlag GmbH & Co. KGaA, Weinheim. (**B**) Schematic illustration of X-ray activated NaCeF_4_:Gd,Tb ScNPs for fluorescence/CT/MR imaging-guided PDT of cancer. Reproduced with permission from Ref. 35, Copyright © 2019, American Chemical Society. (**C**) Construction process of Hb-CPNs@liposome. (**D**) Working model of the luminescing and O_2_-supplying PDT system for cancer treatment. Reproduced with permission from Ref. 36, Copyright © 2019, Wiley-VCH Verlag GmbH & Co. KGaA, Weinheim. Abbreviations: PDT: photodynamic therapy; CT: computed tomography; MR: magnetic resonance; Hb: hemoglobin.

In spite of the popularity of NIR light, its application is still limited by the penetration depth (10-15 mm). As a kind of ionizing radiation source, X-ray has been widely applied in clinical tumor imaging and therapy owning to its unrestricted penetration depth in human body 29. With the above advantage, X-ray can be adopted as a light source to excite PSs. The X-ray-induced PDT (X-PDT) can be categorized as: (1) metal-based X-PDT; (2) rare-earth-element-based X-PDT; (3) quantum dot (QD)-based X-PDT; (4) silicon-based X-PDT 52. For example, Sun *et al.* 34 designed a new PS system (R-AIE-Au) based on rose bengal (RB)-conjugated aggregation-induced emission gold clustoluminogens (AIE-Au) for low-dose metal-based X-PDT. Under X-ray irradiation, the Au atoms efficiently converted X-ray to luminescence and further excited conjugated RB for PDT (**Figure 3A**). *In vitro* and *in vivo* studies verified that R-AIE-Au could generate X-ray-triggered ROS through the unique X-PDT mechanism, thereby realizing the effective treatment of radioresistant cancers. This new PS system possesses the potential for the treatment of deep penetrating tumors. In another study, cerium (Ce)-doped highly fluorescent NaCeF_4_:Gd,Tb scintillating nanoparticles (ScNPs) for rare-earth-element-based X-PDT were reported by Zhong *et al.* 35. The Ce and Tb could absorb the energy of secondary electrons produced by X-ray to generate ROS for significant tumor suppression (**Figure 3B**).

Yang *et al.* 146 proposed a novel QD-Photofrin conjugate for QD-based X-PDT, in which QD (CdSe core with a ZnS shell) was excited by X-ray and transfered energy to conjugated PS *via* fluorescence resonance energy transfer (FRET). The number of visible photons produced by the QD is linearly proportional to the radiation dose. The efficiency of FRET approached 100% when the Photofrin/QD ratio reached 291:1. *In vitro* assays proved that the combination of QD-Photofrin with X-ray resulted in enhanced H460 cell killing when compared with X-ray alone. Rossi *et al.* 147 used SiC/SiOx core/shell scintillating nanowire (ScNW)-conjugated tetra (4-carboxyphenyl) porphyrin (H_2_TCPP) as a new PS system (SiC/SiOx-H_2_TCPP) for silicon-based X-PDT. This SiC/SiOx-H_2_TCPP could generate ^1^O_2_ when exposed to low-dose X-ray irradiation. Moreover, the irradiation only took 20 s, which is shorter than the 40 s and 90 s used in standard clinical treatment.

### Interstitial light

For deeply seated tumors in the body, interstitial PDT (I-PDT) is an alternative treatment which is usually performed by delivering light through multiple cylindrical diffusing fibers inserted into the target tumor. Shafirstein *et al.* 148 employed Photofrin^®^-mediated I-PDT to treat locally advanced head and neck cancers (HNC). I-PDT with Photofrin^®^ significantly improved the cure rate compared to light (630 nm) delivery alone at the same irradiance and light dose. *In vivo* assays proved that local cures of VX2 were obtained in mouse tumors with I-PDT at 16.5-398 mW/cm^2^ and ≥45 J/cm^2^. In addition, the efficient delivery of therapeutic dose to target tumors with minimal damage to nearby intact tissues must be ensured for I-PDT to be used in deep tumors. For this purpose, Ismael *et al.* 149 adopted cylindrical diffuse optical fibers (CDFs) as the light source (630-760 nm) and optimized the output CDFs power to specify the light dose (20-50) J·cm^-2^ to the tumor site, thus achieving the effective treatment of breast cancer. Recently, Vermandel *et al.* 150 applied magnetic resonance imaging (MRI) as an estimator to assess the lesional effect of low-power 5-aminolevulinic acid (5-ALA)-based I-PDT. Based on the observations of MRI data and consideration of therapeutic effects, the 5-fraction light delivered at 5 mW during I-PDT is the most efficient treatment scheme with limited toxicity to adjacent healthy brain tissues, and might be a good way to comprehensively explore cell death pathways based on I-PDT.

### Internal light

Considering that the external light source is limited by the light delivery efficiency, the research focus of light source in PDT further shifts from external light source to internal light source. For instance, Jiang *et al.* 36 prepared a PDT system by using hemoglobin (Hb)-linked conjugated polymer nanoparticles (CPNs) for the internal activation of PS. Specifically, in the presence of H_2_O_2_, Hb catalyzed the activation of luminol to produce chemiluminescence which could be absorbed by CPNs through chemiluminescence resonance energy transfer (CRET) to sensitize O_2_, thereby generating ROS for cancer treatment (**Figure 3C and 3D**). In another example, Sun *et al.* 37 fabricated a luminescence PersLum material hydrogel (PLM-hydrogel) for high-efficient persistent luminescence-sensitized PDT. The PLM-hydrogel was synthesized by dispersing high-temperature calcined PLM into a biocompatible alginate-Ca^2+^ hydrogel. It could be easily injected into tumor sites of mice as a powerful localized light source to trigger continuous ^1^O_2_ generation. *In vivo* tests effectively proved the persistent luminescence, light renewability, and strong fixing ability of PLM-hydrogel in tumors.

### Summary of light source

In a short conclusion, for wider application of PDT, the availability, cost, and delivery efficiency of light source need to be considered, but the first priority is light penetration depth. Present innovative strategies based on NIR light, X-ray and internal light have solved this problem to a certain extent. Nevertheless, the low energy associated with NIR light, the weak PS activation ability and obvious side effects to normal tissues of X-ray, as well as the unsatisfactory energy transfer from internal light to PS remain challenging tasks. Recently, several new PDT excitation sources, including microwaves 130, radio-waves 131, ultrasound 132, electrical fields (EF) 133, and magnetic fields (MF) 134, have emerged and made some progress. Microwave irradiation can cause local hyperthermia and increase the blood supply to the tumor. Ultrasound can penetrate deep into the tumor to activate PSs. The electroporation effect of EF can help PSs pass through the cell membrane. MF can realize the magnetic target accumulation of PSs. Future study of the above excitation sources is encouraged.

## Photosensitizers (PSs)

PSs play a critical role in the therapeutic effect of PDT. Ideally, the most suitable PSs for PDT are adjustable amphiphilic reagents in biological environments. These PSs should exhibit strong molar absorption of long-wavelength light and be non-toxic when not exposed to light. They should also have a long triplet lifetime and an excellent ^1^O_2_ quantum yield 38-40. Ideal PSs should target tumor tissues with long-term retention and then can be rapidly removed from the body after the end of treatment. A great deal of work has been carried out in recent years to develop various PSs for PDT. In this part, we will first introduce the types of PSs, and then discuss the innovative research to enhance ROS generation ability, solubility and targeted ability of PSs.

### Types of PSs

In the last few decades, a large number of organic and inorganic PSs used in PDT have been developed for cancer treatment. The organic PSs used in clinical or preclinical applications of PDT includes porfimer sodium, 2-(1-hexyloxyethyl)-2-devinyl pyropheophorbide-a, verteporfin, temoporfin, silicon phthalocyanine 4, porphyrin, redaporfin, talaporfin, photolon (chlorin e6 (Ce6) sodium-polyvinylpyrrolidone), hemoporfin, padeliporfin, photodithazine, photosens, hiporfin, photocyanine, and radachlorin 30. Hematoporphyrin derivative (HpD), dihaematoporphyrin ether (DhE), and photofrin porfimersodium are the first generation organic PSs widely used for PDT 41. Although they have been approved by the Food and Drug Administration (FDA), their production and efficacy is limited by the complex synthesis process, immature purification technology, and low molar extinction coefficient 42-46. As a result, the second generation PSs with high ^1^O_2_ yields are developed, which includes phthalocyanines (zinc phthalocyanine (ZnPc)) 47, bacteriochlorins 48, hypericin 49, phenothiazines (methylene blue (MB) and toluidine blue) 50, cyanines (merocyanine-540) 51, metalloporphyrins 29, xanthenes (rose Bengal (RB)) 53, and porphycenes 54. Nevertheless, the clinical use of these PSs is also hampered by the targeting and stability issues. To enhance the tumor targeting ability, third generation PSs conjugated with targeted agents are synthesized, such as Ac-L-Phe-ALA-OMe, galactose substituted Si(IV)-phthalocyanine and suger conjugated chlorins 55. In addition, a number of new PSs have emerged, including indocyanine dyes (indocyanine green (ICG), IR-825, and IR-780), BODIPYs, diketopyrrolopyrrole (DPP), aggregation-induced emission (AIE) dyes, curcumin, perylenequinone, furocoumarin, nobel metal complexes (Ru(II)/Ir(III)/Au(III) complexes), organic frameworks compounds (MOFs, covalent organic frameworks (COFs), hydron-bonded organic frameworks (HOFs)), and polymer-based PSs (polyfluorene, polythiophene) 151. However, the targeting efficiency or *in vivo* selectivity of them is not high enough for clinical use. Hence, greater efforts should be devoted to designing PSs with strong stability and targeted ability.

In recent years, increasing attention has been paid to the research of inorganic PSs based on nanomaterials. Generally, nano-inorganic PSs contain gold nanoparticles 56, silver nanoparticles 57, porous silicon nanoparticles 58, CdTe quantum dots (QDs) 59, carbon materials 60, black phosphorus (BP) 61, titanium dioxide (TiO_2_) 62, transition metal oxides (TMOs) heterojunction 63, Ti_3_C_2_ nanosheets 64, and graphitic-phase carbon nitride (g-C_3_N_4_) nanosheets 65. Compared with organic PSs, nano-inorganic PSs are more potent for PDT for several reasons. First, most inorganic PSs are stable even under laser irradiation. Second, the modification and functionalization of inorganic PSs endow them with targeting ability, which is beneficial to the realization of precise cancer treatment and reduction of side effects. Last, some inorganic PSs are widely utilized as carriers of photothermal reagents and chemotherapeutic drugs, allowing for the synergistic treatment of multiple therapeutic approaches. However, the inherent disadvantages of inorganic PSs such as low ^1^O_2_ quantum yield, poor biodegradability and biosecurity largely hinder their clinical application. More attempts are therefore needed to address these limitations.

### Enhanced stability and targeting ability of PSs

**Figure 4:**
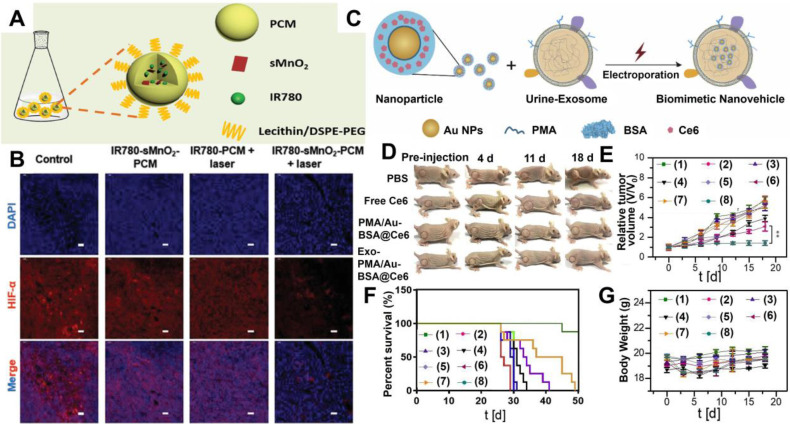
(**A**) Synthetic schematic of IR780-sMnO_2_-PCM nanoparticles. (**B**) Immunofluorescence staining of HIF-1α under different conditions. Reproduced with permission from Ref. 66, Copyright © 2019, Wiley-VCH Verlag GmbH & Co. KGaA, Weinheim. (**C**) Schematic illustrations of the preparation of Exo-PMA/Au-BSA@Ce6 nanovehicles for targeted PDT. (**D**) Tumor growth of mice after different treatments for 18 days. (**E**) Tumor volumes curves, (**F**) survival rates, and (**G**) body weight of mice after different treatments: (1) PBS; (2) PBS+laser; (3) free Ce6; (4) free Ce6+laser; (5) PMA/Au-BSA@Ce6; (6) PMA/Au-BSA@Ce6+laser; (7) Exo-PMA/Au-BSA@Ce6; (8) Exo-PMA/Au-BSA@Ce6+laser. Reproduced with permission from Ref. 69, Copyright © 2019, Elsevier. Abbreviations: PCM: phase change materials; Exo: exosomes; PMA: amphiphilic polymer; Ce6: chlorin e6; PDT: photodynamic therapy.

Most organic PSs are highly hydrophobic and tend to aggregate in aqueous environments. The aggregation process can reduce the therapeutic efficiency of PDT since PSs must remain in monomer form to be photoactive 151. This monomer structure can be achieved by combining PSs with nanomaterials. For example, covalent bonding with hydrophilic polymer molecules can effectively enhance the bioavailability of hydrophobic porphyrins 152. Thus, using nano-sized carriers to deliver PSs can be a feasible strategy to improve the poor stability of traditional organic PSs. Zhang *et al.* 66 utilized thermal responsive phase change materials (PCM) as carriers to co-encapsulate ultrasmall manganese dioxide (sMnO_2_) and organic PSs (IR780) for tumor hypoxia-modulated PDT. The protective PCM layer not only effectively prevented the photodegradation of IR780 but also endowed IR780 with better solubility than free IR780 (**Figure 4A**). Meanwhile, the PCM layer could immediately release sMnO_2_ under laser irradiation, which further triggered the decomposition of endogenous H_2_O_2_ to generate enough O_2_ for relieving tumor hypoxia (**Figure 4B**). *In vivo* photoacoustic (PA) and fluorescence imaging illustrated that IR780-sMnO_2_-PCM nanoparticles (NPs) could accumulate in tumors and showed efficient tumor retention under intravenous injection. As revealed by *in vivo* PDT treatments, IR780-sMnO_2_-PCM NPs displayed superb performance in inhibiting tumor growth.

Recently, naturally derived nanocarriers, including exosomes (Exos) and platelets, have been attracting more attention than other nanocarriers and applied to therapeutics owning to their transcellular permeability and biocompatibility 67,68. For example, Pan *et al.* 69 adopted high-purity urinary Exos to load amphiphilic polymer (PMA)-coated/Au-BSA@Ce6 NPs. In this Exo-PMA/Au-BSA@Ce6 system, the presence of Exos successfully improved the hydrophobicity and poor biocompatibility of Ce6, resulting in enhanced PDT performance with abundant ^1^O_2_ generation. Meanwhile, this Exo-based nanocarrier could penetrate through the membrane barrier and target tumor tissues, thus achieving targeted PDT with deep penetration and superior tumor retention (**Figure 4C-4G**).

**Figure 5:**
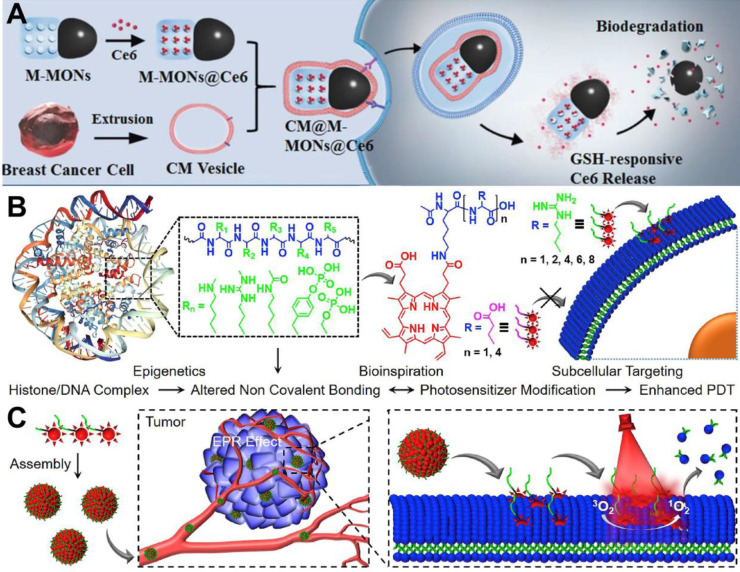
(**A**) Schematic illustration of the preparation of CM@M-MON@Ce6 for PDT. Reproduced with permission from Ref. 70, Copyright © 2019, Wiley-VCH Verlag GmbH & Co. KGaA, Weinheim. (**B**) Chemical structures of amino acids modified PpIX and schematic illustration of plasma membrane-targeted PDT. (**C**) Tumor targeted delivery of Ac-K(PpIX)-En for PDT-induced plasma membrane rupture. Reproduced with permission from Ref. 71, Copyright © 2019, Elsevier. Abbreviations: CM: cell membrane; Ce6: chlorin e6; PDT: photodynamic therapy; PpIX: protoporphyrin IX.

In terms of tumor targeting, surface modification of PSs with targeting ligands or moieties can effectively achieve tumor-targeted accumulation of PSs. Wang *et al.* 70 employed cancer cell membrane (CM) to enhance the tumor-targeted accumulation of PSs. Specifically, PS (Ce6) was embedded with magnetic mesoporous organosilica nanoparticles (M-MONs) and then covered with breast cancer cell membrane (CM@M-MON@Ce6) for PDT (**Figure 5A**). The homologous targeting effect caused by the affinity between the adhesion molecules on the breast cancer cell membrane and their source cells facilitated the favorable tumor-targeted accumulation of CM@M-MON@Ce6 with prolonged blood circulation time. The therapeutic efficacy of CM@M-MON@Ce6 was evaluated by animal experimentation, and the results illuminated that this PDT nanoplatform possessed a remarkable eradication effect on primary tumors.

In another strategy, Cheng *et al.* 71 proposed an amino acid modification strategy to regulate the intracellular distribution of PS (protoporphyrin IX (PpIX)) for targeted PDT. In this platform, the membrane anchoring ability and phototoxicity of PpIX to different cell lines could be effectively controlled at the level of single arginine (**Figure 5B** and **5C**). Systematic studies indicated that the obtained nanoplatform (Ac-K(PpIX)-En) possessed excellent biocompatibility and targeting ability. *In vitro* and *in vivo* assays confirmed the robust anti-tumor effect of Ac-K(PpIX)-En with low biotoxicity. In addition, glycopolymers can also be used as targeting ligands. Sun *et al.* 153 developed a conjugated polymer-based PS (PPF-Ir-*g*-(POEGMA-*b*-PGal)), in which a PS Ir (III) complex was covalently attached to the conjugated backbone (poly(benzene-*alt*-fluorene), PPF-Ir), and a water-soluble polymer (POEGMA-*b*-PGal) was further grafted from the PPF-Ir. The brush structured PPF-Ir-*g*-(POEGMA-*b*-PGal) exhibited excellent water solubility and targeting ability since glycopolymer polygalactose (PGal) can specifically bind to asialoglycoprotein receptor (ASGPR) overexpressed on the surface of HepG2 cells. In an *in vitro* assay, this polymer-based PS damaged HepG2 cancer cells under white light irradiation. Moreover, xenograft HepG2 tumors were significantly inhibited *in vivo*.

### Enhanced ROS generation efficiency of PSs

In addition to improving the stability and targeting of PSs, further advancements on ROS generation efficiency are still required. ^1^O_2_ generation quantum yield can be used as an index to evaluate the photodynamic effect of type II-based PSs. To optimize the ROS generation of PSs, ISC rate constant can be increased by incorporating heavy atoms (N, Br, Pt, Hf, thiophenyl group, etc.) or 2,2,6,6-tetramethylpiperidinyloxy (TEMPO) to PSs, reducing the energy gap (ΔE_ST_) between S_1_ and T_1_, or introducing electron-withdrawing/-donating groups in the conjugated structure 29. For example, Zhou *et al.* 154 combined Ru(II) polypyridyl complex with Pt(II)-based supramolecular coordination complexes to form heterometallic metallacycle Ru-Pt for NIR-activated PDT. The obtained Ru-Pt exhibited a high ^1^O_2_ generation quantum yield (89%) owning to the heavy atom effect of Pt. *In vivo* assays confirmed that Ru-Pt could effectively inhibit tumor growth under low light dose irradiation. In addition to the heavy atom effect, TEMPO can also increase the ISC rate through radical-triplet pair mechanism. By incorporating TEMPO into Cy7 dyes, the as-prepared dye compound (dye 2) displayed a higher ^1^O_2_ generation quantum yield than Cy 7 (20% vs 0.6%) 155. A low ΔE_ST_ can lead to a high ISC rate and promote the energy transfer from ^3^PS* to O_2_, thus decreasing the ΔE_ST_ of PS can be an efficient way to increase ^1^O_2_ quantum yield. Shi *et al.* 156 successfully synthesized two DPP derivatives, phenyl-substituted DPP (PDPP) and thienyl-substituted DPP (TDPP). The ΔE_ST_ values of PDPP and TDPP are calculated to be 0.66 and 0.48 eV, respectively. Both *in vitro* and *in vivo* experiments proved that TDPP performed better in generating ^1^O_2_ to kill cancer cells and eliminate tumor than PDPP. In addition, the electron-withdrawing and electron-donating groups can affect the energy level of complexes and facilitate the ISC process. For example, a coumarin-modified Ru(II) PS (Ru2) shows lower oxidation potential and higher extinction coefficient than a coumarin-free Ru(II) PS, resulting in improved ROS generation efficiency 118. A series of push-pull AIEgens synthesized by coupling diphenylamine group with different electron-withdrawing groups on carbazolyl rings also exhibit enhanced ^1^O_2_ generation capability 157. For the semiconductor nanomaterials, the ROS generation efficiency can be improved by optimizing their size, morphology, heteroatom doping, and surface state.

### Summary of PSs

In brief, a great many of PSs for PDT have been developed over the last few decades. The latest knowledge indicates that the modification of PSs using nanocarriers and targeting conjugates can significantly enhance their water-solubility, targeting and delivery efficiency. In addition to phase change materials, exosomes, cancer cell membrane and amino acids, some commonly used nanocarriers and targeting conjugates in other strategies are summarized in Table 1. Nevertheless, it is still complicated and challenging to improve the stability, solubility, and tumor targeted ability of PSs since their morphology, particle size, and surface modification can significantly affect the stability and tumor-targeting ability. In addition, it is worth noting that targeting is an extensively studied but unproven concept that has been around for a long time, so it may be unlikely to translate into clinical practice. More efforts are needed in the research of PSs to facilitate the clinical application of PDT.

## Oxygen dependence

Most existing PDT systems are in the form of type II mechanism that involves significant O_2_ consumption. However, inadequate O_2_ concentration is a characteristic feature of many solid tumors, which significantly hampers the antitumor effect of PDT 98. To deal with tumor hypoxia, great efforts have been made in recent years and many innovative strategies have been proposed. Based on the mechanisms responsible for PDT process, these innovative strategies can be classified into three categories: (1) O_2_-replenishing strategies that can increase tumor O_2_ concentration by direct or indirect means; (2) O_2_-independent strategies that can perform PDT even under low O_2_ conditions; (3) O_2_‑economized strategies that can block intracellular O_2_ consumption. In this section, we will discuss in detail these three categories of PDT innovative strategies dedicated to overcoming tumor hypoxia.

### O_2_-replenishing strategies

#### Delivery of O_2_ into the tumor

**Figure 6:**
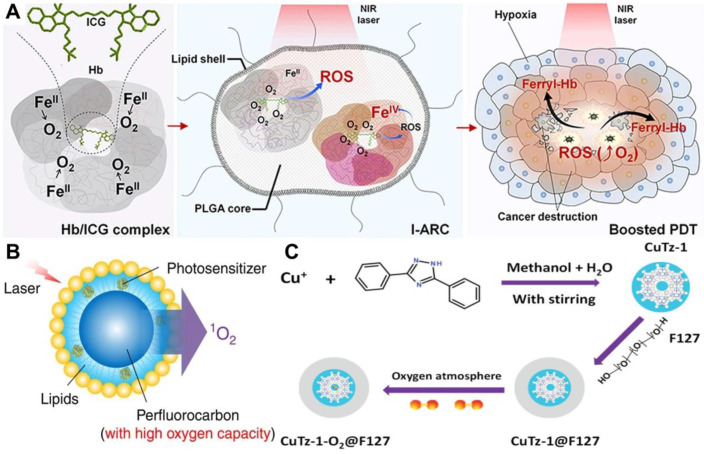
(**A**) Schematic illustration of PS system based on Hb and ICG co-loaded RBCs for tumor-boosted PDT. Reproduced with permission from Ref. 100, Copyright © 2016, Nature Publishing Group. (**B**) Schematic of PFCs internalized Oxy-PDT agent for tumor inhibition. Reproduced with permission from Ref. 103, Copyright © 2015, Nature Publishing Group. (**C**) Schematic of the synthetic procedure of CuTz-1-O_2_@F127 for enhanced PDT. Reproduced with permission from Ref. 105, Copyright © 2019, Wiley-VCH Verlag GmbH & Co. KGaA, Weinheim. Abbreviations: Hb: hemoglobin; ICG: indocyanine green; RBCs: artificial red blood cells; PDT: photodynamic therapy; PFCs: perfluorocarbons.

Direct delivery of O_2_ into tumor with appropriate O_2_ carriers is one of the most common strategies to overcome tumor hypoxia in PDT. Commonly used O_2_ carriers include Hb, perfluorocarbons (PFCs) and MOFs. The O_2_ carrying capacity of Hb makes it an attractive candidate for reoxygenation. Considering the poor stability and short circulation time of Hb, artificial red blood cells (RBCs) are combined with Hb to conquer the defects of free Hb 99. For example, Luo *et al.* 100 fabricated artificial RBCs to load Hb and ICG for O_2_-supply PDT. Similar to natural RBCs, these Hb-contained artificial RBCs could reversibly bind to four O_2_ molecules to form HbO_2_ (**Figure 6A**), thus enabling the stable self-enrichment of O_2_ for subsequent massive ROS generation. Importantly, Hb could be oxidized into highly oxidative ferryl-Hb species, leading to the synergistic oxidative damage of xenograft tumor. Other Hb-related O_2_ carrier systems have also been investigated, such as liposomes and polymeric micelles 101. However, since each Hb molecule can only bind to four O_2_ molecules, these systems are limited to a certain extent by O_2_ loading ability.

To achieve a high O_2_ delivery, PFCs can be an excellent choice thanks to the high electronegativity of fluorine atoms that endows PFCs with excellent O_2_ affinity. Consequently, PFCs are utilized as important components of O_2_ delivery systems to surmount hypoxia and improve the effectiveness of PDT in cancer therapy 102. Cheng *et al.* 103 developed a novel O_2_ self-enriched PDT system (Oxy-PDT) by loading a PS (IR780) into PFCs nanodroplets (**Figure 6B**), in which a sufficient O_2_ concentration was maintained. Both *in vitro* and *in vivo* tests confirmed the enhanced therapeutic efficacy of Oxy-PDT, which was attributed to the high O_2_ availability for ^1^O_2_ generation in Oxy-PDT. Moreover, the half-life of ^1^O_2_ in PFCs was 5 × 10^-2^ s, which is much longer than that in the cellular environment (6 × 10^-7^ s) or water (5 × 10^-6^ s), resulting in long-lasting PDT effect. Similarly, Zhang *et al.* 158 synthesized a poly(ethylene glycol)-boron dipyrromethene amphiphile (PEG-F_54_-BODIPY) with 54 fluorine-19 (^19^F), and employed it to emulsify perfluorohexane (PFH) into a nanoemulsion (PFH@PEG-F_54_-BODIPY). The as-prepared nanoemulsion significantly enhanced the therapeutic effect of BODIPY by dissolving O_2_ and reducing the self-quenching of BODIPY molecules. *In vitro* and *in vivo* assays indicated that PFH@PEG-F_54_-BODIPY) could effectively alleviate tumor hypoxia through the O_2_ storage capability of PFH.

MOFs are a new kind of porous nanomaterials with ultrahigh surface areas and uniform pore sizes. The use of MOFs as O_2_ storage/carrying nanomaterials for hypoxia modulation has been extensively investigated 104. Recently, Gao *et al.* 80 utilized the significant gas-storage capability of zirconium (IV)-based MOFs (UiO-66) to realize O_2_-evolving PDT. As a carrier for O_2_ storing, UiO-66 was conjugated with PS (ICG) through coordination reaction and further encapsulated in RBCs membranes to construct a PDT nanoplatform (O_2_@UiO-66@ICG@RBC). The O_2_ isotherms demonstrated that the O_2_ storage capacity of UiO-66 reached 500 μmol g^-1^ and the stored O_2_ was effectively released under NIR light irradiation. Owing to the long circulation and O_2_ self-sufficiency of MOFs, the designed nanoplatform displayed an enhanced efficiency of tumor ablation. In another work, Cai *et al.* 105 designed a biodegradable O_2_-loaded MOF therapeutic platform (CuTz-1-O_2_@F127) (**Figure 6C**). The O_2_-loaded CuTz-1@F127 could accumulate in the tumor due to the enhanced permeability and retention (EPR) effect and release the absorbed O_2_ at the tumor site. The high antitumor efficacy of this O_2_-evolving PDT platform was successfully demonstrated by *in vitro* and *in vivo* studies.

#### *In situ* O_2_ generation

There are three strategies to produce O_2_ *in situ*: (1) H_2_O_2_-decomposition triggered O_2_ generation; (2) water-splitting mediated O_2_ generation; (3) photosynthetic O_2_ production. For the first way, considering the high H_2_O_2_ level in TME, *in situ* O_2_ generation inside tumor by catalyzing the decomposition of H_2_O_2_ can be an effective strategy to overcome tumor hypoxia 106. For the second mode, inspired by the nature, scientists have developed photocatalytic reactions to generate O_2_ from H_2_O 107. For the third manner, it is generally believed that O_2_ molecules come from primitive photosynthesis of microorganisms (cyanobacteria and archaea) 108, which can photosynthesize to produce O_2_ using the photosystem on the thylakoid membrane.

**Figure 7:**
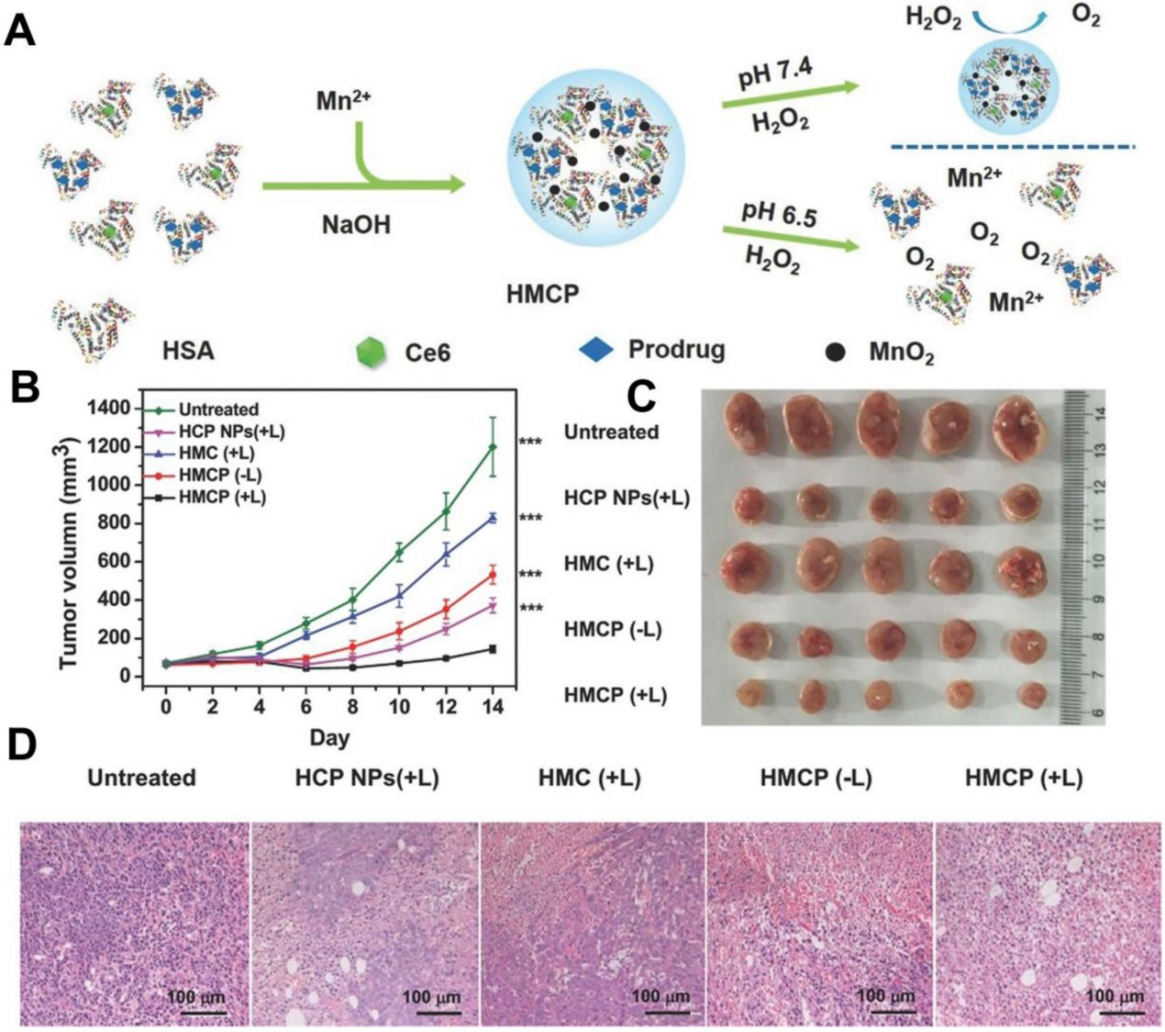
(**A**) Schematic illustration of the fabrication of HSA-MnO_2_-Ce6&Pt nanoparticles. (**B**) Tumor volume curves of mice on day 14 after various treatments, and (**C**) corresponding photographs of tumors collected from mice at the end of treatment. (**D**) H&E staining assay of tumor slices from different groups of mice collected 24 h after 661 nm light irradiation. Reproduced with permission from Ref. 110, Copyright © 2016, Wiley-VCH Verlag GmbH & Co. KGaA, Weinheim. Abbreviations: Ce6: chlorin e6; H&E: hematoxylin and eosin.

In the H_2_O_2_-decomposition triggered O_2_ generation strategy, catalase 109 and inorganic nanomaterials based on calcium (Ca), gold (Au), platinum (Pt), manganese (Mn), Ce and copper (Cu) are widely used to catalyze H_2_O_2_ decomposition in recent years 106. For instance, Chen *et al.* 110 constructed an intelligent H_2_O_2_-responsive and O_2_-evolving nanosystem based on MnO_2_ nanoparticles (HSA-MnO_2_-Ce6&Pt NPs) to overcome the tumor hypoxia-associated resistance of PDT. In this nanosystem, MnO_2_ could generate O_2_ *in situ* by reacting with endogenous H_2_O_2_ inside TME (**Figure 7**). Yao *et al.* 111 proposed synergetic mesoporous cerium oxide upconversion nanoparticles (Ce-UCNPs) to achieve the pH/H_2_O_2_-responsive self-sufficiency of O_2_. Cerium oxide NPs can reversibly switch from Ce^4+^ to Ce^3+^ and simultaneously catalyze H_2_O_2_ to O_2_ due to their enzyme-like activity 112. *In vivo* tests demonstrated that the Ce-UCNPs could efficiently decompose the intracellular H_2_O_2_ to generate sufficient O_2_, successfully overcoming hypoxia. Another strategy for designing O_2_-supply PDT platforms is to use Pt NPs. Liang *et al.* 81 prepared a nano-multifunctional platform (PDA-Pt-CD@RuFc) by loading Pt-modified and cyclodextrin (CD)-decorated polydopamine (PDA) NPs with a Ruthenium (II) complex (RuFc). The Pt NPs catalyzed the generation of O_2_ from H_2_O_2_, and then RuFc photoactivated O_2_ to produce ^1^O_2_ for PDT. This nanoplatform successfully overcame the tumor hypoxia.

**Figure 8:**
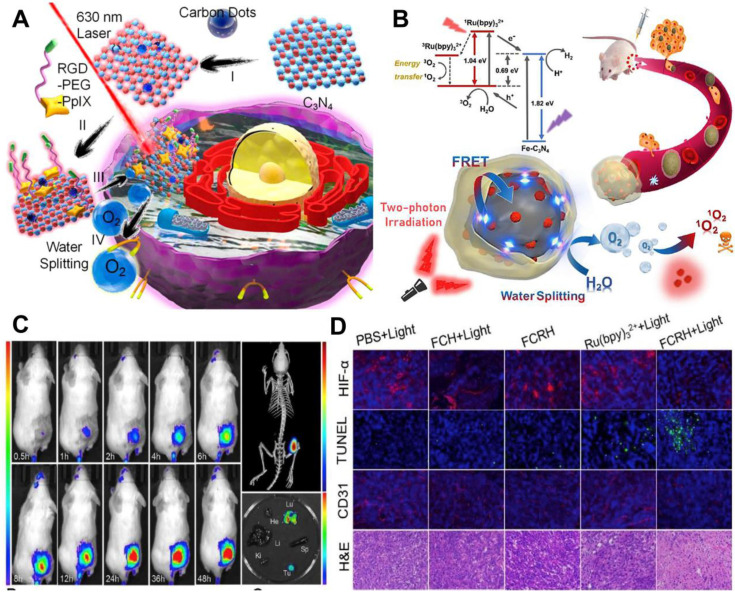
(**A**) Schematic illustration of PCCN-mediated water splitting enhanced PDT. Reproduced with permission from Ref. 107, Copyright © 2016, American Chemical Society. (**B**) Scheme diagram of the two-photon excited Fe-C_3_N_4_@Ru@HOP (FCRH) nanocomposite for efficient PDT against hypoxic tumor. (**C**) Fluorescence imaging and micro-CT transillumination fluorescent combination imaging (top right) of FCRH *in vivo*, and its *ex vivo* fluorescence imaging in heart, liver, spleen, lung, kidney and tumor (bottom right). (**D**) HIF-α, TUNEL, CD31 and H&E staining assays of tumors after various treatments. Reproduced with permission from Ref. 113, Copyright © 2018, Elsevier. Abbreviations: PDT: photodynamic therapy; CT: computed tomography; H&E: hematoxylin and eosin.

For water-splitting mediated O_2_ generation strategy, carbon nitride (C_3_N_4_)-based multifunctional nanocomposite (PCCN) was prepared by Zheng and co-workers 107 for light-driven water splitting, in which carbon dot-doped C_3_N_4_ was assembled with PS (PpIX) *via* π-π stacking. In this nanosystem, the water-splitting efficiency of C_3_N_4_ alone is limited, but the use of carbon dots to enhance the red light absorption could improve its water-splitting efficiency under 630 nm laser irradiation, ultimately increasing the intracellular O_2_ concentration. Subsequently, PpIX transmitted the energy to the produced O_2_ to generate ^1^O_2_ under a 630 nm laser (**Figure 8A**). *In vitro* and *in vivo* assays indicated that this PCCN nanosystem possessed excellent capability to improve the intratumoral O_2_ level. Compared with 630 nm laser, NIR can penetrate into deeper tissues with lower scattering and absorption. On this basis, Li *et al.* 113 reported iron-doped C_3_N_4_ (Fe-C_3_N_4_) and the ability of water-splitting mediated O_2_ generation was also demonstrated. Ru (Ⅱ) complex (Ru(bpy)_3_^2+^) as a PS was loaded onto Fe-C_3_N_4_ to form Fe-C_3_N_4_@Ru@HOP (FCRH) nanocomposite. Since the Ru (Ⅱ) complex could be excited by simultaneous absorption of two photons of NIR light, a two-photon laser was used to activate O_2_ production through Fe-C_3_N_4_ and ^1^O_2_ generation by Ru(bpy)_3_^2+^ to provide appropriate synchronization for the two processes (**Figure 8B-8D**).

**Figure 9:**
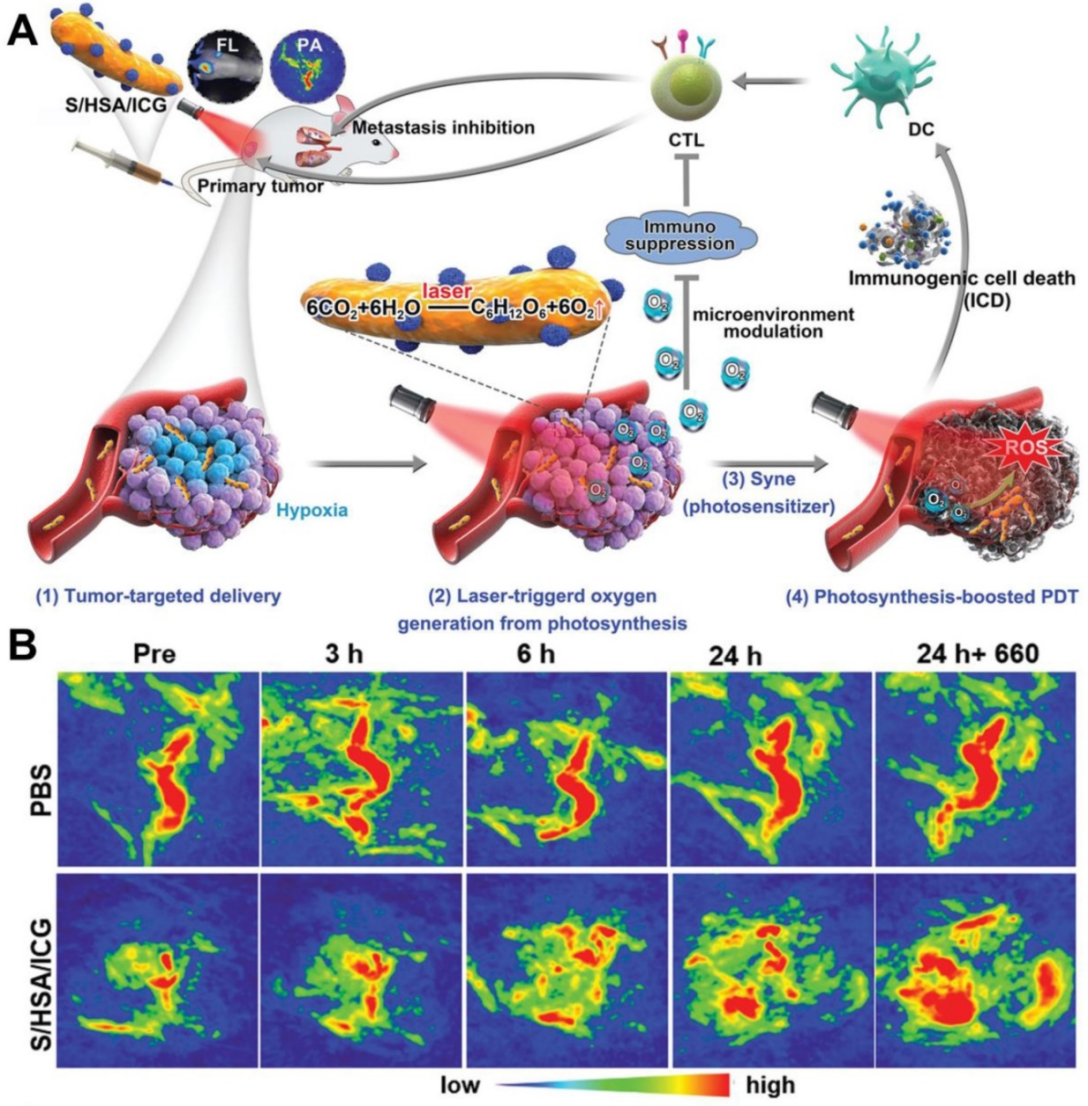
(**A**) Schematic illustration of S/HSA/ICG as an O_2_ generation system for photosynthesis-boosted PDT. (**B**) PA images of tumors taken at different time points after intravenous injection of PBS and S/HSA/ICG. Reproduced with permission from Ref. 116, Copyright © 2020, Wiley-VCH Verlag GmbH & Co. KGaA, Weinheim. Abbreviations: ICG: indocyanine green; PDT: photodynamic therapy; PA: photoacoustic.

In the photosynthetic O_2_ production strategy, microorganism-based PDT for hypoxic tumor therapeutics was reported 114. Huo *et al.* 115 fabricated a photosensitive and photosynthetic cyanobacteria-based PDT system (ceCyan cells) by encapsulating PSs (Ce6) inside the *Synechococcus elongatus* PCC 7942 (Cyan). Under 660 nm laser irradiation, the ceCyan cells generated O_2_ through photosynthesis, and Ce6 activated the O_2_ into ^1^O_2_. The cascade process could produce large amounts of ^1^O_2_ to destroy cancer cells both *in vitro* and *in vivo* with a single-wavelength laser light source. Considering that photosynthetic bacteria (*Synechococcus* 7942, Syne) possess stronger tumor targeting ability than Cyan, Liu *et al.* 116 utilized Syne as the PS carrier and *in situ* photocatalyzed O_2_ generation to realize photosynthesis-boosted PDT (**Figure 9A**). ICG-encapsulated NPs (HSA/ICG) were assembled and then connected with the surface of Syne *via* amide bonds to form a PDT system (S/HSA/ICG). The photoautotrophic Syne not only promoted the delivery of ICG into the tumor through its intrinsic targeting ability, but also increased the intratumoral O_2_ concentration through photosynthesis under the irradiation of 660 nm laser, thus improving the therapeutic efficacy of PDT. Upon intravenous injection, S/HSA/ICG effectively accumulated in the tumor site and generated abundant O_2_ continuously under laser irradiation (**Figure 9B**), which remarkably ameliorated tumor hypoxia and boosted ROS production. It should be pointed out that although the above researches have effectively demonstrated the ability of photosynthetic bacteria to producce O_2_ *via* photosynthesis, the O_2_ generation efficiency has not been studied, and more efforts are needed.

### O_2_-independent strategies

**Figure 10:**
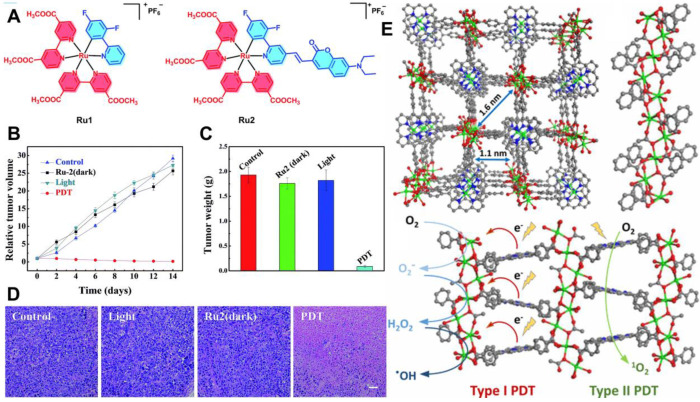
(**A**) Chemical structures of Ru1 and Ru2. (**B**) Tumor volume curves of mice after various treatments, and (**C**) corresponding tumor weights after 14 days of treatment. (**D**) H&E staining of tumor slices from different groups. Reproduced with permission from Ref. 118, Copyright © 2018, Royal Society of Chemistry. (**E**) Structure of Ti-TBP and schematic diagram of type I and type II PDT under light irradiation. Reproduced with permission from Ref. 119, Copyright © 2019, American Chemical Society. Abbreviations: H&E: hematoxylin and eosin.

Apart from O_2_-replenishing strategies, new paradigms involving diminished O_2_ dependence are also favored in PDT. As described above, type I mechanism is an electron transfer process utilized in PDT systems. It can produce toxic free radicals including ·OH and ·O_2_^-^ under laser irradiation. Studies show that type I PDT performs well even at low O_2_ concentrations, which lays foundation for designing new approaches to break through the hypoxia limitation of type II PDT 117. In a recent work by Lv *et al.* 118, a coumarin-modified cyclometalated Ru(II) PS (Ru2) exhibited excellent type I PDT effect. The successful modification of Ru(II) complex by coumarin enhanced the light-absorption ability of Ru2 in the visible region (400-800 nm). The PDT effect was evaluated under both normoxia and hypoxia conditions, and the results showed that Ru2 maintained superior PDT activity even under hypoxia (**Figure 10A-10D**), which could be attributed to the direct electron transfer between the excited Ru2 and the adjacent substrates through the photochemical process of type I.

In another work, Lan *et al.* 119 demonstrated a new MOF-based PDT system (Ti-TBP) consisting of Ti-oxo chain secondary building units (SBUs) and 5,10,15,20-tetra (p-benzoato) porphyrin (TBP, a kind of PSs), which could be used for hypoxia-tolerant type I PDT. Upon 650 nm light irradiation, Ti-TBP transferred electrons from excited TBP* to Ti-oxo chain SBUs to produce TBP^•+^ and Ti^3+^ species, which induced the generation of ·OH and ·O_2_^-^ (**Figure 10E**), and thus elicited superb anticancer effect of type I PDT with a tumor suppression rate of 98%. Similarly, Bevernaegie *et al.* 120 developed photo-oxidizing iridium(III)-based sensitizers (Ir(III)) to damage hypoxic tumors through type I mechanism under oxygen-free conditions. Viability assays were performed on 3D tumor spheroids under both normoxic and hypoxic conditions, and the corresponding results proved that Ir(III) could penetrate deeply into 3D tumor spheroids and destroyed them completely under 405 nm light irradiation even at low O_2_ concentrations.

Recently, Li *et al.* 121 proposed a nanostructured 2,4,6-tris-(N,N-dimethylaminomethyl)phenoxy substituted Zinc(II) phthalocyanine assembly (NanoPcA) that could trigger ROS generation *via* type I mechanism. In this PDT system, the NanoPcA possessed high electron-donating ability which is conducive to promoting type I photoreactions. Moreover, the electron-rich surface of NanoPcA caused by amine groups in TEG facilitated highly efficient ROS generation under 655 nm laser irradiation. Therefore, NanoPcA can serve as a PS to hypoxic tumor PDT through type I photoreactions. In addition to the visible light-activated type I PDT mentioned above, NIR-activated type I PDT is also developed for deep tissue penetration depth. Li and co-workers 122 engineered tungsten carbide nanoparticles (W_2_C NPs) to produce ·OH and ^1^O_2_ *via* type I and type II reaction mechanisms for NIR-activated dual-type PDT. The synthesized W_2_C NPs exhibited strong light absorption in NIR II region and could simultaneously generate ·OH and ^1^O_2_ merely under 1064 nm laser irradiation. In addition, the excellent photothermal activity of W_2_C NPs allowed synergetic therapeutic effects of PDT/PTT. *In vitro* and *in vivo* tests confirmed the convenient and highly-efficient anti-tumor capability of W_2_C NPs even in hypoxic tumors.

### O_2_‑economized strategies

Different from the O_2_-replenishing strategies and O_2_-independent strategies mentioned above, O_2_-economized strategies are targeted to regulate tumor hypoxia by decelerating cell respiration. It is noticed that the mitochondria-associated oxidative phosphorylation (OXPHOS) process can lead to excessive physiological O_2_ consumption which is well known as cell respiration 123. Scientists believe that the block of O_2_ consumption in OXPHOS by inhibiting the activity of Complex Ⅰ in the mitochondrial electron transport chain (ETC) would be a feasible and meaningful strategy to relieve tumor hypoxia 124. In one study, Yang *et al.* 125 synthesized a tailor-made PDT platform (PEG-PCL-IR780-MET NPs) by packaging PSs (IR780) and metformin (MET) in poly (εcaprolactone)-poly (ethylene glycol) (PEG-PCL). Once these PEG-PCL-IR780-MET NPs accumulated in tumor tissues, IR780 could generate ROS under 808 nm laser irradiation, and MET could directly inhibit the activity of Complex Ⅰ in mitochondrial ETC, thus achieving superior PDT efficacy.

**Figure 11:**
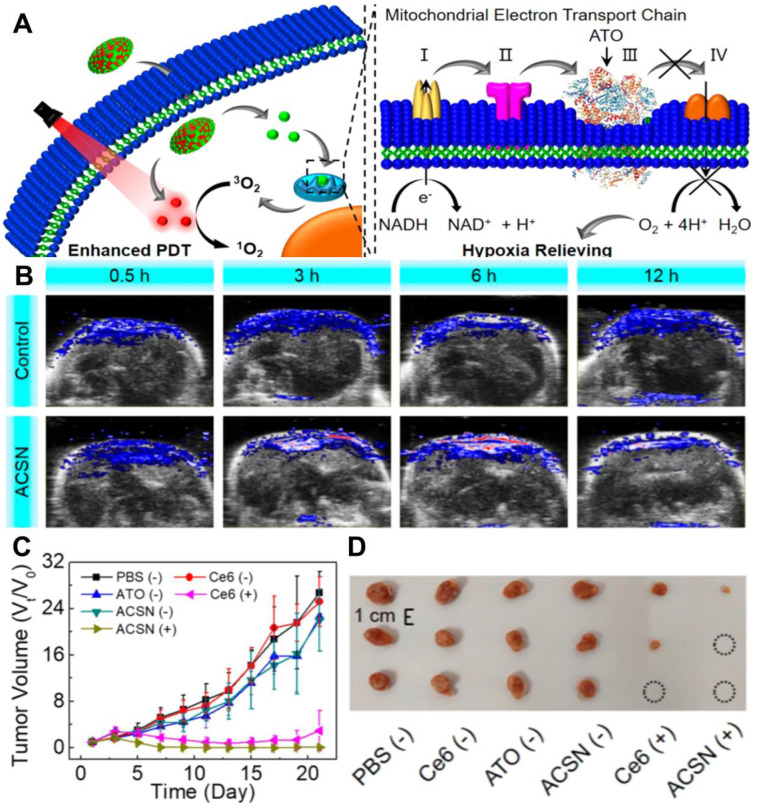
(**A**) Schematic diagram of mitochondrial respiratory inhibition enhanced PDT against tumor cells. (**B**) *In vivo* PA images of tumors after various treatments for 0.5, 3, 6, and 12 h. (**C**) Corresponding tumor volume changes of mice during the 21 days evaluation period with various treatments, and (**D**) corresponding tumor images at 21st-day post-treatment. Reproduced with permission from Ref. 126, Copyright © 2020, American Chemical Society. Abbreviations: PDT: photodynamic therapy; PA: photoacoustic.

In another work, Zhao *et al.* 126 prepared atovaquone (ATO, an oxidative phosphorylation inhibitor) and Ce6-based self-delivery nanomedicine (ACSN) for O_2_‑economized PDT. With a high drug loading rate, ACSN significantly improved the stability of Ce6 and ATO, and enhanced cell internalization and intratumor permeability as well. The role of ATO in this work is to inhibit the mitochondria respiratory chain, reduce O_2_ consumption and relieve hypoxia, thereby increasing the production of ROS and improving the efficiency of PDT (**Figure 11A**). As a result, ACSN exhibited robust inhibition of tumor growth with low systemic toxicity (**Figure 11B-11D**). To further enhance the penetration of drugs into the tumor, Fan *et al.* 127 established sub-50 nm dual-drug nanoparticles (NPs) to encapsulate verteporfin (VER, a kind of PSs) and oxygen-regulator ATO (ATO/VER NPs) to attenuate hypoxia-induced resistance to PDT. In this system, ATO/VER NPs could penetrate into the interior regions of the tumor. Then, the ATO released from ATO/VER NPs efficiently reduced cellular oxygen consumption by blocking the ETC pathway and further heightened VER to generate ^1^O_2_. Both* in vitro* and *in vivo* tests proved the powerful anti-tumor PDT effect of ATO/VER NPs.

In addition, Li *et al.* 128 investigated the influence of antiestrogenic drug tamoxifen (TAM) on mitochondria respiration. They successfully devised a ·O_2_^-^ generator (SORgenTAM) on the basis of TAM. Results of a series of tests confirmed that TAM could disturb the energy metabolism of cells by inhibiting mitochondrial Complex Ⅰ in ETC. Consequently, TAM significantly overcame the hypoxia resistance in PDT. In addition to MET, ATO and TAM, nitric oxide (NO) also possesses the ability to inhibit cellular respiration and disturb cell metabolism. Yu *et al.* 129 fabricated a PDT-specific O_2_ economizer by using poly(D,L-lactide-co-glycolide) nanovesicles (PVs) to co-load NO donor (sodium nitroprusside (SNP)) and tetraphenylporphyrin (TPP)). Once the TPP and SNP co-loaded PVs (PV-TS) accumulated in tumors through EPR effect, the released SNP could respond to locally reductive environment and decompose to generate NO that could compete with O_2_ by binding to the O_2_-binding site of mitochondria for respiration inhibition. As a result, such a PV-TS system showed outstanding antitumor performance toward hypoxic tumors.

### Summary of innovative strategies for overcoming tumor hypoxia

In this section, different innovative strategies for mitigating the hypoxic tumor microenvironment have been summarized. In general, the number of research publications on hypoxia regulation has grown exponentially. Most of these studies present encouraging results in overcoming tumor hypoxia and ultimately improving cancer therapy. However, we should be cautious about the application of these emerging strategies to circumvent hypoxia, since they are still in the early stage of development and require more rigorous testing before clinical trials.

## Topical PDT

In clinical trials to date, PSs are usually administered through intravenous injection, oral or topical delivery. Topical PDT is a non-invasive and rapidly evolving therapeutic form to treat precancerous cutaneous lesions or non-melanoma skin cancers, such as basal cell carcinoma (BCC), squamous cell carcinoma *in situ*, and actinic keratosis 159. The topical PS precursors, aminolevulinic acid (ALA) and its derivatives (methyl aminolevulinate (MAL) and hexyl aminolevulinate (HAL)) are not photosensitive but can be converted endogenously into PpIX by the haem biosynthesis pathway. PpIX that tends to accumulate in malignant cells can be activated by blue or red light to generate ROS, causing selective cellular damage 160. The advantage of topical PDT is it can treat multiple lesions simultaneously with excellent tolerance and superior cosmetic outcomes.

ALA (a 20% 5-ALA solution in alcohol) is commercially available and FDA approved for the treatment of non-hyperkeratotic actinic keratosis on the face and scalp in combination with blue light (417 nm) 161. In Europe, a gel formulation of 10% ALA in nano-emulsion was developed and licensed by FDA to treat actinic keratosis under the excitation of a red light (635 nm) LED lamp 162. Considering that ALA is a water-soluble amino acid with low lipid solubility and cannot penetrate the stratum corneum, MAL (a 16% MAL topical cream) was used to treat BCC and actinic keratosis in combination with a red light lamp (570-670 nm) 163. The above three ALA-/MAL-based topical PDT drugs have been tested in clinical studies for several indications. For example, in a phase III trial involving 88 patients with BCCs, ALA PDT showed shorter healing time and better cosmetic outcomes compared to cryosurgery (12-month clinical recurrence rate of 5% versus 13%) 164. Similarly, a multicenter randomized controlled trial among 196 patients revealed the excellent therapeutic effect of MAL PDT for superficial BCCs (complete response (CR) rate at 3 months: 92.2%, 12-month recurrence rate: 9.3%) 165. In another phase III comparative trial, 281 patients with BBCs were treated with 10% ALA gel and MAL cream. Effective PDT was observed in both formulations with CRs of >90% 166.

## PDT-involved multimodal therapies

Complete eradication of malignant tumors or effective prevention of the metastasis and relapse by PDT alone is difficult due to the inherent defects of PDT. The combination of PDT with other therapies can exploit the advantages of each therapeutic modality to offset the disadvantages of PDT. The interaction between different therapies can not only produce synergistic therapeutic effects, but also improve the anti-tumor efficacy at low-dose PSs or low-power light irradiation, thereby minimizing the potential toxicity to normal tissues. Several combination therapy partners for PDT, such as chemotherapy and immunotherapy, have been approved in initial clinical studies 30. This section will focus on the research progress of chemo-photodynamic therapy (chemo-PDT) and immuno-photodynamic therapy (immuno-PDT).

### Chemo-PDT

The combination of PDT and chemotherapy can induce synergistic therapeutic effects: the PSs can overcome multidrug resistance, while the chemotherapeutic drugs can address the limitations of light penetration and hypoxia-related resistance in PDT and enhance the sensitivity of cancer cells to ROS. Recently, Zhang *et al.* 167 reported an albumin 'nanoglue'-based nanotheranostics for chemo-photodynamic combination therapy, which consists of HSA, chemotherapeutic drug paclitaxel (PTX), and photosensitizer sinoporphyrin sodium (DVDMS). The as-prepared HSA-PTXDVDMS nanoparticles (HPD) exhibited good stability and effective accumulation in tumors after intravenous injection. Particularly, the HPD showed enhanced antitumor efficacy compared to DVDMS alone, displaying the broad clinical application prospects of chemo-PDT. Similarly, Wang *et al.* 168 constructed an intelligent protoporphyrin-based polymer nanoplatform with a multiple stimuli-responsive function for combined chemo-PDT. These polymer micelles (Dex-g-PpIX-g-PBA-SS-CPT (DPPSC)) were composed of dex-tran (Dex), PpIX, anticancer drug camp-tothecin (CPT), and pH-sensitive linker. Once the micelles enter the tumor, the photochemical internalization (PCI) effect of PpIX could facilitate cellular uptake, and the CPT could be released from the micelles by the hydrolysis of pH-sensitive linker to achieve chemotherapy. The combined chemo-PDT is expected to maximize the therapeutic effect and minimize the side effects of chemotherapeutic agent.

Preclinical and clinical studies have shown that PDT combined with chemotherapy is an effective tumor treatment option 175. For example, Hong *et al.* 176 compared the long-term curative effect of PDT alone (porfimer sodium) and PDT combined with chemotherapy (gemcitabine or gemcitabine with cisplatin) on patients with cholangiocarcinoma. There was no statistically significant difference in tumor node metastasis (TNM) stage, bismuth type, CA 19-9 level, and pre-PDT albumin level between two groups. However, PDT with chemotherapy resulted in significant improvement of overall survival compared to PDT alone (median survival: 538 days versus 334 days). It was easily concluded that PDT combined with chemotherapy for advanced hilar cholangiocarcinoma is superior to PDT alone. Especially, PDT-mediated vascular permeabilization can enhance the accumulation of drugs in tumors for enhanced efficacy 177. Luo *et al.* 178 used a semi-mechanistic pharmacokinetic-pharmacodynamic (PK/PD) model to investigate the effect of PDT on drug delivery. Long-circulating liposomes loaded with doxorubicin (DOX) and porphyrin-phospholipid (PoP) PSs were administered intravenously to mice. Tumor irradiation with 665 nm laser light (200 J/cm^2^) for 1 h showed increased drug accumulation, as evidenced by an overall 7-fold increase in DOX area under the tumor curve in the PK/PD model. This study adequately demonstrated the enhancement of PDT-based drug delivery.

### Immuno-PDT

The occurrence and metastasis of tumors is highly related to the evasion of immune surveillance system. Various studies have profoundly demonstrated that PDT can cause immunogenic cell death (ICD) to release damage-associated molecules and consequently improve the immunogenicity of tumors 169. Moreover, the combination of PDT with checkpoint-blockade immunotherapy can better eliminate primary tumors, inhibit metastasis, and prevent recurrence 179. For example, Xu *et al.* 180 synthesized UCNPs to simultaneously load Ce6 and imiquimod (R837, a Toll-like-receptor-7 agonist) for immuno-PDT. In tumor experiments, UCNP-Ce6-R837 could control the regrowth of primary tumors under NIR irradiation, while its effect on distant tumors was limited. Anti-CTLA-4 alone decreased tumor growth rate, but did not lead to the regression of primary or distant tumors. UCNP-Ce6-R837 combined with CTLA-4 blockade not only displayed excellent efficacy in eliminating primary tumor but also resulted in strong antitumor immunities to inhibit the growth of distant tumors. Similarly, Zhang *et al.* 170 fabricated an immunoadjuvant nano-agent (γ-PGA@GOx@Mn,Cu-CDs) by integrating γ-glutamic acid (γ-PGA), glucose oxidase (GOx) and Mn,Cu-doped carbon dots (CDs) with excellent photodynamic effect under 730 nm laser irradiation. After combined with PD-L1 antibody for checkpoint-blockade immunotherapy, γ-PGA@GOx@Mn,Cu-CDs significantly ablated primary tumors and suppressed distant tumors through antitumor immune response. In another work, Sun *et al.* 171 successfully developed a sorafenib and Ce6 co-loaded ROS-responsive nanoparticle (NP-sfb/ce6) for combined immuno-PDT. Under 660 nm laser irradiation, the Ce6-produced ROS destroyed the nanoparticles, leading to the boosted cascade release of sorafenib. The low-dose PDT and rapidly released sorafenib synergistically inhibited tumor growth by inducing T cell-dependent antitumor immune responses and reprograming the immunosuppressive TME. Thus, the combination of PDT with immunotherapy has the potential to eradicate tumors and trigger immune memory to prevent tumor recurrence.

## Conclusions and future perspectives

PDT has made dramatic progress over the past few decades because of its feasibility and effectiveness in cancer treatment. Recent advances in nanotechnology have opened promising avenues for developing new PDT systems and provided multifaceted opportunities to circumvent the intrinsic drawbacks in current PDT paradigms. This review has systematically summarized and discussed the latest research development of PDT in light source, PSs, and tumor hypoxia. For light source, we have highlighted the latest innovative strategies based on NIR light, X-ray and internal light. In terms of PSs, we have introduced different types of PSs and the research progress of nanocarriers and targeting conjugates for improving the stability and targeting ability of PSs. As for tumor hypoxia, we have summarized three kinds of innovative strategies to overcome the hypoxic limitation in PDT. Nevertheless, in spite of all the remarkable progress, there still exist some issues in PDT as detailed below.

(i) As described above, a critical challenge for PDT in cancer therapy is the limited penetration depth of light. Visible light is not an ideal choice due to the strong light absorption of tissue. NIR light is limited by the penetration depth of less than 2 cm. Moreover, the energy of NIR light may not be high enough to effectively activate PSs to generate adequate ROS. X-ray can deeply penetrate into tissues, but it exhibits low ROS generation efficiency and can cause side effects on healthy tissues. This technique needs to be improved to achieve better therapeutic effect with low doses of X-ray radiation. Although internal light is not affected by the light delivery efficiency, its photoexcitation efficiency is unsatisfactory. Therefore, an ideal light source is still under exploration.

Recently, researchers have started to utilize microwaves 130, radio-waves 131, ultrasound 132, EF 133, MF 134, and electromagnetic fields (EMF) 135 to induce PDT. Microwave irradiation can cause local hyperthermia and lead to tumor sensitization, which is useful for increasing the blood supply to the tumor and promoting ROS generation. Ultrasound, a type of mechanical wave, can penetrate into cancer targets buried deep in human tissues. It can precisely focus on specific tumor sites and effectively activate the cytotoxicity of PSs, ultimately triggering tumor cell destruction with minimal damage to adjacent normal tissues 172. The electroporation effect caused by EF can help PSs pass through the cell membrane, thus enhancing the PDT effect. MF can promote the accumulation of magnetic PSs in tumorous region. Hence, future research is encouraged to study more about the above excitation modes. Additionally, light radiation conditions such as exposure time and pulse frequency should be taken into consideration.

(ii) The therapeutic effect of PDT is largely related to the choice of PSs that are expected to possess strong tumor targeting ability, excellent stability, and high ROS generation efficiency. Since the morphology, particle size, and surface modification can significantly affect the stability and targeting efficiency of PSs, a lot of efforts are needed to develop ideal PSs that can be activated by long wavelengths with high ROS yields. The application of nanomaterial agents in PDT can overcome some shortcomings of conventional PSs. The combination of PSs and magnetic nano-emulsion may be a new strategy to realize targeted MF-induced PDT. Moreover, transition metal complexes can also be a choice to construct PSs with tumor-targeting ability and high ROS generation efficiency. For example, some tris-chelated Ir^III^ complexes exhibit long-lived triplet excited states and phosphorescence, which allows them to generate destructive ^1^O_2_ efficiently even under hypoxic conditions 136. In addition, it is also very important to choose the appropriate concentration and administration mode of PSs.

(iii) As mentioned earlier, tumor hypoxia can significantly hamper the antitumor effect of PDT. Although many innovative strategies including O_2_-replenishing, O_2_-independent, and O_2_‑economized strategies have been put forward to deal with this issue, there are still several issues need to be concerned: 1) how to quantify the O_2_ supply level of O_2_-replenishing strategies for PDT; 2) how to obtain biocompatible hypoxia-confronted PDT systems without obvious toxicity; 3) how to transform the innovative PDT agents for hypoxic tumor elimination into clinical applications. In addition, great efforts can also be made to develop other new strategies. For instance, the recently emerged strategies to improve blood flow, reduce ROS scavenging, remodel extracellular matrix, and combine with hypoxia-activated therapeutic modalities have also become effective approaches to overcome tumor hypoxia 137-139.

In spite of the aforementioned challenges, underlying mechanism issues (e.g. pharmacokinetic/pharmacodynamic analysis) are also crucial in determining the optimal conditions for PDT and ensuring the long-term biosafety of PDT agents. Moreover, it is worth noting that the currently used tumor models are simplified. They are rather different from the actual situation. In addition, for the multifunctional theranostic nanomaterials currently proposed for PDT, a comprehensive understanding of the way they behave as a collective entity and affect complex biological systems is still lagging behind. Admittedly, none of these nanomaterials has been approved by FDA for clinical application. Therefore, it is urgent to conduct in-depth characterization of them to check whether they will interfere with each other's functions and impede the overall therapeutic effect. In this regard, standardized physicochemical characterization and evaluation protocols must be established to achieve the regulatory review of nanotechnology-based PDT agents. Undoubtedly, there is still a long way to go before PDT become a first-line treatment option for cancer. It is hoped that this review would advance future development of PDT and provide encouraging possibilities for effective cancer therapy.

## Figures and Tables

**Table 1:** Some commonly used nanocarriers and conjugates for PDT

Nanocarriers	Loaded PSs	Ref.	Conjugates	Targated tumor/cancer cells	Ref.
SiO2 NPs	Purpurin-18, HpD, hypocrellin A, chlorin, MB, PHPP and ZnPc	16,72	Breast cancer cell membrane	Breast cancer cells	70
Liposomes	m-THPC	74	Arginine	Plasma membrane	71
Micelles	PpIX	75	Folic acid	Cell surface folate receptors	88
Carbon nanomaterials	ICG, Ce6, porphyrin	76,79	Phenylalanine	Transformed PAM212 keratinocyte cells	89
Peptide-Based nanoparticles	Phthalocyanine	77,141	Retinoic acid	Neuroblastoma SK-N-DZ cells surface retinoic acid receptors	90
Bacteriophage nanowires	Pyropheophorbide a	78	Glucose and galactose	K562 cells	91
Polymer	Ce6, MB, m-THPC, PpIX, graphitic carbon nitride quantum dots	81,98	Low-density lipoprotein	Colon, HepG2 and retinoblastoma tumors	92
Exosomes	Ce6	67,69	Magnetoliposomes	Melanoma	93
Phase-change materials	IR780	66	Epidermal growth factor	Breast carcinoma cells, epidermal carcinoma and ovarian cancer	94
LDHs	ZnPc, Ce6, ICG	33,83	Monoclonal antibodies	Ovarian and breast cancer cells	95
MOFs	Ce6, ICG	80,84	Human serum albumin	Breast cancer cells	96,140
2D transition metal dichalcogenides (TMDs)	Ce6, MB	85,173	RGD (Arg-Gly-Asp)	HepG2	112
Mesenchymal stem cells	Purpurin-18	73	Triethylene glycol (TEG)	Breast cancer cell surface protein	123
MnO_2_	Silicon phthalocyanine dihydroxide, Ce6, TiO_2_	86,174	(4-carboxybutyl)triphenylphosphonium bromide	Mitochondria	97
			Hyaluronic acid (HA)	Cell surface CD44 receptor	63
			Glycopolymer	The asialoglycoprotein receptor (ASGPR) overexpressed on the surface of HepG2 cells	153

## Abbreviations

PDT: photodynamic therapy
PSs: photosensitizers
ROS: reactive oxygen species
^1^O_2_: singlet oxygen
·O_2_^-^: superoxide radicals
·OH: hydroxyl radicals
^1^PS*: excited singlet state
^3^PS*: triplet state
O_2_: oxygen
NIR: near-infrared
MOFs: metal-organic frameworks
UCNPs: upconversion nanoparticles
GQD: graphene quantum dot
IPA: isophthalic acid
RTP: room-temperature phosphorescence
LDHs: layered double hydroxides
RB: rose bengal
AIE: aggregation-induced emission
Hb: hemoglobin
CPNs: conjugated polymer nanoparticles
CRET: chemiluminescence resonance energy transfer
PLM-hydrogel: PersLum material hydrogel
HpD: hematoporphyrin derivative
DhE: dihaematoporphyrin ether
FDA: Food and Drug Administration
ZnPc: zinc phthalocyanine
Ce6: chlorin e6
MB: methylene blue
BP: black phosphorus
TiO_2_: titanium dioxide
TMOs: transition metal oxides
g-C_3_N_4_: graphitic-phase carbon nitride
PCM: phase change materials
sMnO_2_: ultrasmall manganese dioxide
PA: photoacoustic
NPs: nanoparticles
Exos: exosomes
PMA: amphiphilic polymer
CM: cancer cell membrane
M-MONs: magnetic mesoporous organosilica nanoparticles
PpIX: protoporphyrin IX
TMDs: transition metal dichalcogenides
TEG: triethylene glycol
HA: hyaluronic acid
PFCs: perfluorocarbons
RBCs: artificial red blood cells
UiO-66: zirconium (IV)-based MOFs
TME: tumor microenvironment
Ca: calcium
Au: gold
Pt: platinum
Mn: manganese
Cu: copper
CD: cyclodextrin
PDA: polydopamine
RuFc: Ruthenium (II) complex
Cyan: *Synechococcus elongatus* PCC 7942
Syne: *Synechococcus* 7942
TBP: 5,10,15,20-tetra (p-benzoato) porphyrin
W_2_C NPs: tungsten carbide nanoparticles
OXPHOS: mitochondria-associated oxidative phosphorylation
ETC: electron transport chain
MET: metformin
ATO: atovaquone
VER: verteporfin
TAM: tamoxifen
NO: nitric oxide
PVs: poly(D,L-lactide-co-glycolide) nanovesicles
SNP: sodium nitroprusside
TPP: tetraphenylporphyrin
EF: electrical fields
MF: magnetic fields
EMF: electromagnetic fields
